# The efficacy of multisite MRI scanners for total brain volume measurements: a cross-sectional study in Saudi Arabia

**DOI:** 10.3389/fradi.2026.1818230

**Published:** 2026-06-17

**Authors:** Magbool Alelyani, Moawia Gameraddin, Ahmad Joman Alghamdi, Sultan Alamri, Nahla Faizo, Sahal Alotaibi, Mona Alghamdi, Samirah Althomali, Nawaf Khabrani

**Affiliations:** 1 Department of Radiological Sciences, College of Applied Medical Science, King Khalid University, Abha, Saudi Arabia; 2 Department of Diagnostic Radiology, College of Applied Medical Sciences, Taibah University, Al-Madinah Al-Munawwarah, Saudi Arabia; 3 Radiological Sciences Department, College of Applied Medical Sciences, Taif University, Taif, Saudi Arabia; 4 Radiology Department, King Faisal Medical Complex, Ministry of Health, Taif, Saudi Arabia

**Keywords:** 1.5 T and 3 T MRI scanners, brain volumetry, multisite imaging, scanner variability, neuroimaging, FreeSurfer

## Abstract

**Background:**

Accurate estimation of brain structure and volume is essential in multi-scanner MRI studies investigating neurological condition. This study examines the effect of cross-scanner differences, incorporating field strength, vendor, and sequence variations, on brain volumetric measurements in Saudi adults.

**Objective:**

to evaluate the agreement, reliability and the magnitude and direction of the systematic bias of volumetric measurements between two MRI systems (1.5 T and 3 T) differing in field strength, vendor, and sequence using a within-subject same-day design.

**Material and methods:**

38 healthy participants (25.8 ± 5.6 years; 52.6% men) were scanned back-to-back on 1.5 T GE OPTIMA 450W (SPGR) at 3 T Siemens Skyra (MPRAGE). The images were analyzed using FreeSurfer v7.1.1 (longitudinal stream). To evaluate mean-level bias, a series of paired t-tests, percent differences, and false-discovery-rate (FDR) – adjusted *p*-values were computed. To evaluate order rank reliability, we used Pearson correlations, and two-way mixed-effects intraclass correlation coefficients [ICC(3,1)].

**Results:**

The global volume measurements were nearly the same on both scanners (total brain: 1 185 ± 99 mL at 1.5 T vs.1 179 ± 103 mL at 3 T; −0.6% mean difference, d = 0.07; FDR-*p* = 0.93) with excellent reliability (ICC = 0.993). The volumes of cortical GM, total GM, supratentorial, and cerebral WM showed a bias < 1.8% with excellent ICCs ≥ 0.978. On the contrary, some subcortical structures had a strong effect of cross-scanner differences after FDR correction: putamen (+13.1%, d = 1.26), amygdala (+10.1%, d = 0.95), accumbens (+36.1, d = 1.58), and thalamus (−6.6%, d = 0.70). Nevertheless, reliability of these nuclei remained good to excellent (ICC = 0.82−0.95). Some of the corpus-callosum subregions showed mixed directional changes (central: −22.0%; anterior: +8.6%) but had good reliability (ICC = 0.76−0.93).

**Conclusion:**

There is high reliability and agreement for whole-brain, cortical, and most subcortical measures between the two MRI scanners under controlled acquisition and standardized processing conditions. Systematic, region-specific biases, especially in the putamen, amygdala, accumbens, and thalamus, necessitate harmonization prior to pooling multisite data.

## 1 Introduction

Brain volume measurements play a critical role in understanding neurological diseases and monitoring treatment response (1). High-resolution Magnetic Resonance Imaging (MRI) provides the optimal modality for visualizing anatomical changes in the brain (2). However, differences in magnetic field strengths—particularly between 1.5 T and 3 T systems—may introduce variability due to differences in tissue contrast, signal-to-noise ratio (SNR), and magnetic susceptibility effects (2). With the growing of large multi-center neuroimaging studies, and the establishment of data sharing consortia, there is an increased need to integrate MRI data sets collected at varying field strengths. While previous studies have reported the reliability of global brain volumetric measurements across multiple scanners, there are still critical gaps in our understanding of the interchangeable volumetric measurements of specific regions, and of the subcortex and deep grey matter in particular. Field strength related variability is especially problematic for studies of neurodegeneration, aging, and disease in data pooled from multi-site studies. Additionally, the analytical frameworks employed have been restricted to small sample sizes and excluded the Middle East, which renders prior findings lacking in external validity. Therefore, there is an urgent need to bridge this gap by evaluating volumetric measurement agreement and scanner effect bias due to variations in field strength to establish harmonization frameworks and facilitate integrated neuroimaging studies to address health disparities in different populations.

An ongoing question in neuroimaging research is whether data acquired at different magnetic field strengths can be used interchangeably, particularly when estimating brain structure volumes. Prior research shows that even repeated scans on the same scanner yield variability (ICC ≈ 0.8) (3, 4) and even lower ICC values for cortical thickness (ICC ≈ 0.5) (5). Thus, scanner differences may affect reproducibility.

Studies comparing different field strengths have reported varying observations. Heinen et al. reported nearly identical measurements (<0.1% difference) across scanners for total brain volume, gray matter, and white matter (6). Conversely, Chu et al. found more substantial differences in subcortical regions (7). Most previous studies used small sample sizes (<20), limiting statistical power and generalizability (6, 7). Recent studies further elucidate the variability in harmonizing data across different cross-scanner imaging and harmonization challenges. A study conducted by Buchanan et al. evaluated measures obtained from structural MRIs on 1.5 T and 3 T systems (8). They reported high reliability for global brain volumes, coupled with systematic variances across structural measures. Additionally, a recent study by Lee et al. assessed the effects of multisite variances in MRI data acquisition and described the role of harmonization techniques to mitigate differences across MRI vendors in multisite implementations and acquisitions (9). Collectively, findings from these studies demonstrate that, while there is high overall agreement across different MRI systems, structural (e.g., regional volumetric) measures exhibit higher sensitivity to differences in magnet strength, vendor implementation, and data acquisition order. These studies clearly illustrated that cross-study integration of MRI data (across different imaging systems and research sites) requires a high level of standardization and harmonization to mitigate variability associated with sophisticated statistical techniques. To bridge the gap, this study implemented a same-day, within-subject dual-clinical MRI system (1.5 T GE SPGR and 3 T Siemens MPRAGE) with standardized segmentation using FreeSurfer. The Saudi adult cohort bolstered population-based coverage in a geographic region where the cross-scanner neuroimaging reliability studies are sparse. This study provides evidence from Saudi Arabia to support the use of integrated 1.5 T/3 T volumetric studies while also identifying areas of concern.

The ultimate aim of this study is to meticulously assess the agreement, reliability, and potential systematic bias in volumetric brain measurements acquired from 1.5 T and 3 T MRI scanners within the same group of healthy Saudi adults, with a particular focus on deep-gray matter regions, as these are known to be variable in response to acquisition changes. This study utilizes standardized FreeSurfer-based segmentation and a within-subject, same-day scanning protocol to assess the degree to which volumetric data from varying field strengths can be reliably integrated in multisite and longitudinal neuroimaging studies. The study specifically aims to identify both global and region-specific effects, particularly within subcortical nuclei, thereby guiding best practices for harmonization, data pooling, and interpretation in forthcoming multicenter neuroimaging researches.

## 2 Materials and methods

### 2.1 Study design

This study was designed as a prospective, within-subject, cross-sectional investigation intended to evaluate the agreement and reliability of volumetric brain measurements between two MRI scanners with varying magnetic field strengths. All participants underwent imaging on both a 1.5 T and a 3 T MRI scanner during a single study visit, allowing each individual to serve as their own control. This method reduces variability among individuals and enhances the statistical power to identify scanner-related differences. The study complied with STROBE (Strengthening the Reporting of Observational Studies in Epidemiology) guidelines for cross-sectional studies and was executed following recognized neuroimaging reliability protocols.

### 2.2 Participants

46 healthy adult volunteers were first recruited from the local community at King Faisal Medical Complex, Taif, Saudi Arabia. Volunteers informed consent was obtained in accordance with the Declaration of Helsinki, and the study protocol was approved by the Ministry of Health, Directorate of Health Affairs - Taif Governorate Institutional Review Board (IRB registration HAP-02-T-067; approval number 502).

All individuals underwent extensive screening before they were given the opportunity to enlist. Neurological or psychiatric conditions, prior cardiac surgery, migraines, claustrophobic, and people who have metallic implants or dental braces that may potentially cause MRI artifacts are excluded. 8 subjects were excluded for the following reasons: cardiac surgery, (*n* = 2), migraine history, (*n* = 3), (*n* = 1) claustrophobic, and (*n* = 2) dental braces, leaving a total of 38 participants (mean age 25.8 ± 5.6 years; age range 18–39 years). None of the included participants had any MRI contraindications, and none showed signs of neurological impairment. In order to ensure direct comparability and minimize biological variability, participants were set to undergo both 1.5 T and 3 T MRI scans on the same day, with the order of the scans being counterbalanced across subjects.

This within-subject design enabled all participants to act as their own control, which maximized statistical power and minimized possible inter-individual confounds. Basic demographic information such as age and sex was recorded for all participants. The cohort consisted of male and female volunteers, most of whom were right-handed; detailed demographic information is provided in Table 1.

**Table 1:** The structural T1-weighted imaging parameters.

Imaging parameters	Siemens scanner	GE scanner
Field strength	3T	1.5T
Protocol	MPRAGE	SPGR
Voxel size (mm^3^)	1.0 × 1.0 × 1.0	1.0 × 1.0 × 1.0
Field of view FOV (mm)	256 (256 × 256 matrix)	256 (256 × 256 matrix)
Slices orientation	176 sagittal slices (3D)	176 sagittal slices (3D)
Slice thickness (mm)	1	1
Repetition time (ms)	2,530	2,730
Inversion time (ms)	1,100	1,000
Echo time (ms)	1.64	3.5

In order to further control for potential confounds, study participants were asked to keep their hydration, sleep, and activity levels consistent on the day of the scan. Prior to each MRI session, safety screenings were repeated, and participants were guided through a standard comfort protocol to prevent motion and other artifacts. None of the participants experienced any adverse events and all of them completed both MRI sessions.

### 2.3 Imaging protocol

All participants were scanned on both MRI scanners on the same day: - GE OPTIMA 450W (1.5 T; SPGR sequence) - Siemens MAGNETOM Skyra (3 T; MPRAGE sequence). The protocols were optimized per FreeSurfer guidelines (10), as shown in Table 1. Imaging parameters included 1.0 × 1.0 × 1.0 mm voxel size, 176 sagittal slices, and consistent field-of-view settings.

### 2.4 Image processing

All scans were inspected for artifacts, consistent with recommended quality-control procedures in neuroimaging research (3, 11). DICOM images were converted to NIFTI using MIPAV (v9.0.0). Automated segmentation was performed using “recon-all -i input_image.nii -all” in FreeSurfer v7.1.1, (https://surfer.nmr.mgh.harvard.edu/) (12), using the recommended longitudinal stream (10). Processing hardware and operating system (Ubuntu 20.04.2) were kept constant to prevent software-related variability, as recommended by Gronenschild et al. (11). Outputs were visually checked for misregistration.

### 2.5 Statistical analysis

IBM-SPSS Statistics V.26 and the R programming language (v4.2.0) were used to perform statistical processing, and to gain comparative metrics as well as determine the reproducibility of volumetric measurements with the 1.5 T and 3 T MRI scanners.

Compressed histograms and descriptive statistics (average, dispersion) were created for Volumes of Interest (VOI) for the two different field strengths. This applies to global and regional brain structures. Integrated regional measurements were evaluated using paired t-test. A threshold of *p* < 0.05 was considered statistically significant. To compensate for multiple volumetric measurement discrepancies, the False Discovery Rate (FDR) technique was utilized when applicable. The ROIs in the ASEG segmentation were included in the multiple-comparison correction to avoid bias in the selection process. FDR correction was applied across all 41 ROIs, which were obtained from the FreeSurfer ASEG atlas.

Linear correlation of volumetric measurements was made on the two scanners using the Pearson correlation coefficient (r). Measuring the consistency and reliability of cross-scanner concordance is performed using the intraclass correlation coefficient (ICC). A two-way mixed model of consistency [ICC (3, 1)] and agreement was used, and 95% confidence intervals (95%CI) were calculated. Cicchetti describes ICC values as being insufficient, unremarkable, fair, and good, with values less than 0.40 being poor, 0.40–0.59 being fair, 0.60–0.74 being good, and 0.75 and above being excellent (13). Mean differences and average discrepancies among the two different field strengths of the key structures were assessed using a Bland-Altman Plot. Effect sizes (Cohen's d) and percentage differences were computed for all significant results to measure the practical magnitude of the scanner-related differences. All volumetric outputs were derived from segmentations of the ASEG atlas from FreeSurfer (12). Statistical significance was determined at *p* < 0.05 after FDR correction unless specified otherwise.

### 2.6 Ethical approval

This prospective study was approved by the Ministry of Health, Directorate of Health Affairs – Taif Governorate (IRB registration HAP-02-T-067; approval number 502), in accordance with the Declaration of Helsinki.

### 2.7 Funding

The authors extend their appreciation to the Deanship of Research and Graduate Studies at King Khalid University for funding this work through Large Research Project under grant number RGP2/431/45.

## 3 Results

**Figure 1:**
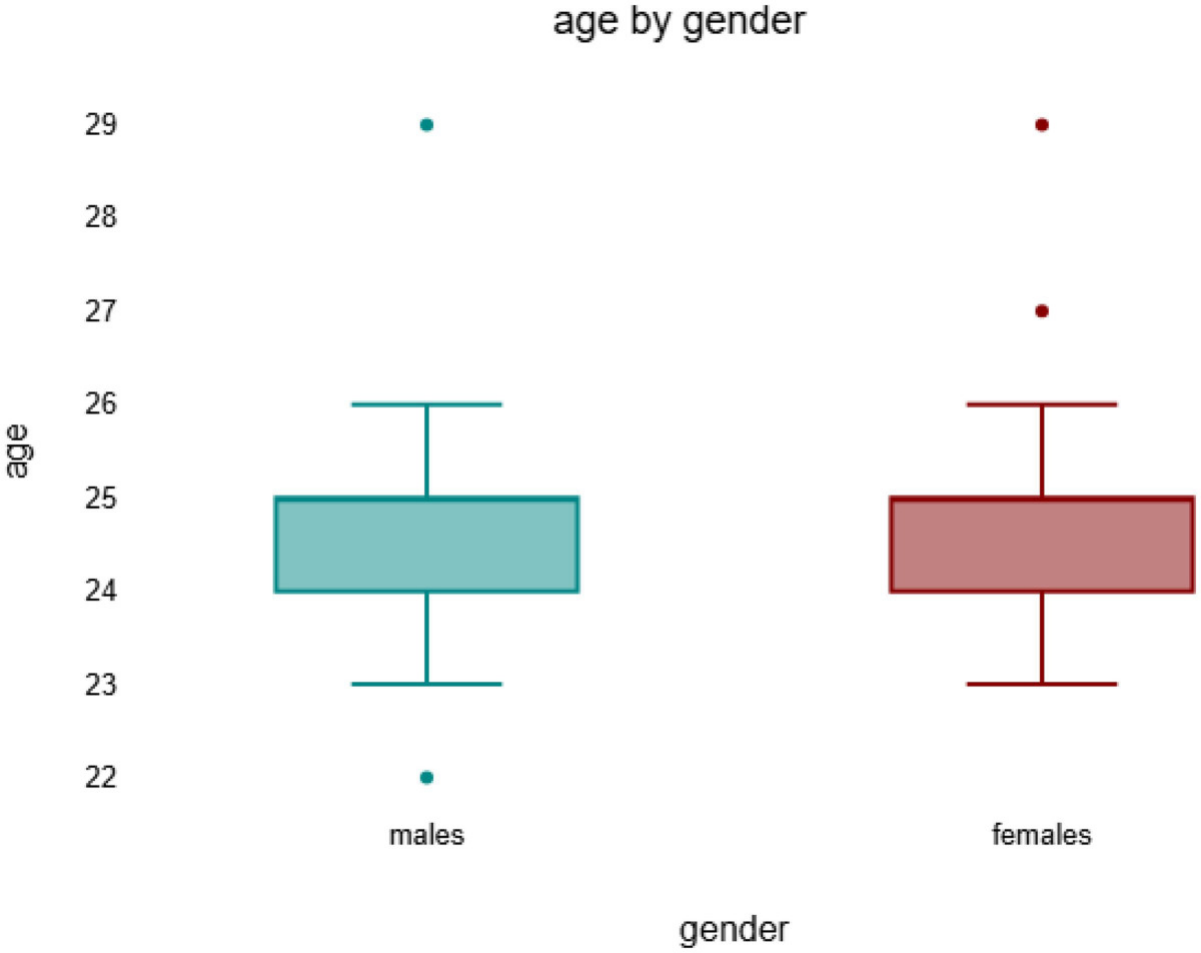
Distribution of age and gender of the participants.

The sample size composed of 21 females and 17 males with mean age of 25 ± 1.47 years (Figure 1). According to FreeSurfer, the results were the measurements of the brain volume (Table 2). These segments included cerebral WM, cerebral cortex, lateral ventricles, third ventricle, fourth ventricle, thalamus, caudate, putamen, pallidum, hippocampus, amygdala, accumbens area, and CSF. All measurements are expressed in cubic millimeters (mm^3^). The volumes from both hemispheres (right and left) were summed to generate a single value for each structure.

**Table 2:** Freesurfer measurements (mean ± STD) with the statistically significant calculation (*p*-value) between 1.5 T and 3 T.

ROI	1.5 T (mean ± SD) mm^3^	3 T (mean ± SD) mm^3^	*p*-value
Total brain volume	1,185,283 ± 99,426	1,178,542 ± 102,826	0.772
Total brain volume (no ventricles)	1,168,488 ± 97,427	1,159,970 ± 100,749	0.709
Supratentorial volume	1,042,712 ± 90,283	1,043,264 ± 95,385	0.979
Supratentorial (no ventricles)	1,025,917 ± 88,366	1,024,692 ± 93,375	0.953
Total gray matter	659,129 ± 50,822	660,305 ± 52,675	0.921
Cerebral white matter	482,189 ± 50,025	473,591 ± 51,254	0.462
Cortex volume	485,827 ± 40,246	490,228 ± 42,894	0.646
Subcortical gray matter	59,848 ± 5,043	62,143 ± 4,986	0.050
Thalamus	16,989 ± 1,601	15,873 ± 1,604	0.003
Caudate	7,053 ± 901	7,340 ± 919	0.174
Putamen	9,865 ± 1,031	11,157 ± 1,039	<0.001
Pallidum	4,113 ± 536	4,117 ± 405	0.970
Hippocampus	8,301 ± 668	8,439 ± 613	0.349
Amygdala	3,142 ± 339	3,459 ± 385	<0.001
Accumbens	997 ± 191	1,359 ± 279	<0.001
Ventral diencephalon	8,329 ± 835	8,408 ± 768	0.668
Corpus callosum (total)	3,928 ± 446	3,689 ± 409	0.017
CC anterior	915 ± 123	994 ± 132	0.008
CC mid-anterior	689 ± 141	583 ± 100	<0.001
CC central	716 ± 119	559 ± 93	<0.001
CC mid-posterior	570 ± 96	541 ± 91	0.178
CC posterior	1,037 ± 146	1,012 ± 172	0.498
CSF	990 ± 225	1,002 ± 260	0.833
3rd ventricle	858 ± 209	984 ± 231	0.015
4th ventricle	1,777 ± 426	1,747 ± 433	0.758
Lateral ventricles	12,351 ± 4,205	13,667 ± 4,349	0.184

### 3.1 Brain volume measurements

**Figure 2:**
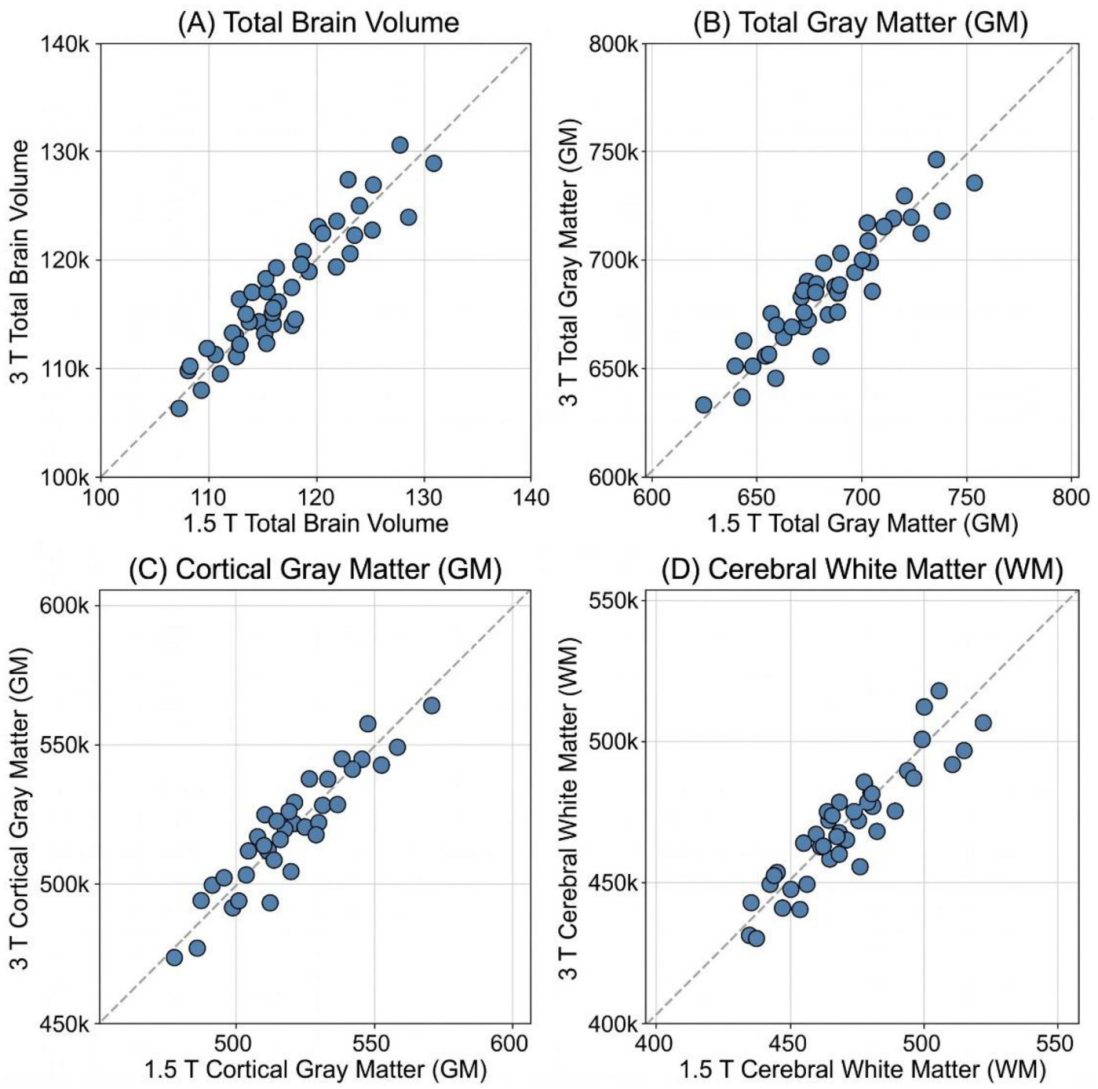
Scatter plots of selected global volumetric measures between 1.5 T and 3 T MRI scanners. **(A)** Total brain volume, **(B)** total gray matter volume, **(C)** cortical gray matter volume, and **(D)** cerebral white matter volume. Each point represents paired measurements from an individual participant. The solid line represents the line of best fit, and the dashed line represents the line of identity (y = x). The strong linear relationships indicate high agreement and consistency between scanners for global volumetric measures.

**Figure 3:**
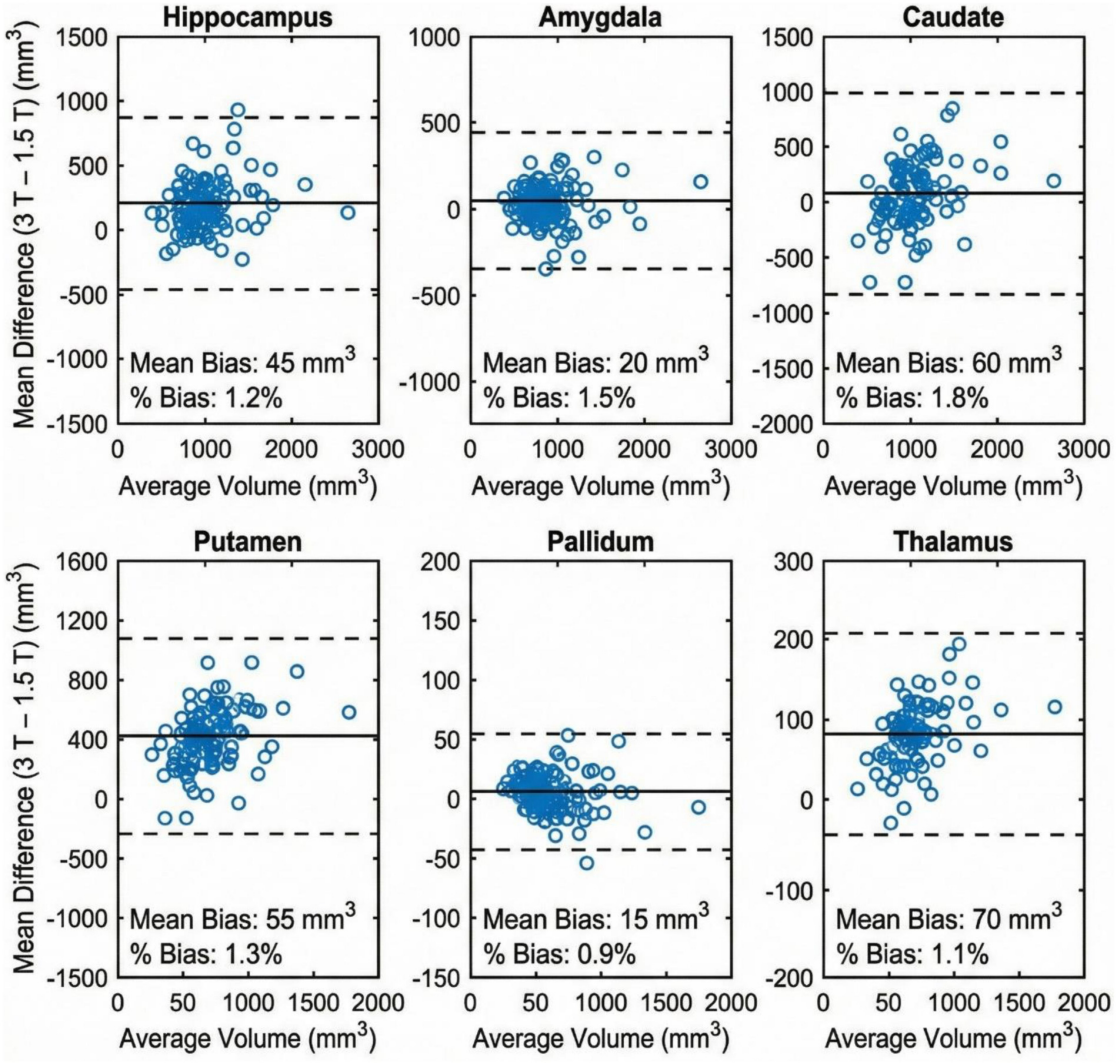
Bland–Altman analysis of selected subcortical volumetric measures between 1.5 T and 3 T MRI scanners. The plots show agreement among scanners for hippocampal, amygdala, caudate, putamen, pallidal, and thalamic volumes. The solid line represents the mean bias between 3 T and 1.5 T measurements, while the dashed lines indicate the 95% limits of agreement. These scatter plots illustrate structure-specific scanner-related differences, particularly in deep gray matter regions.

Analysis of scatter plots demonstrated an extremely strong linear correlation for selected global volumetric measures between 1.5 T and 3 T MRI scanners with Pearson's r of 0.99 and above for total brain, cortical gray matter (GM), and cerebral white matter (WM) (Figure 2). Table 2 shows the descriptive statistics for all structures. Paired t-tests comparing total brain volume, supratentorial volume, GM, WM, or cortical volume showed no significant differences (all FDR-corrected *p* > 0.05; Table 2). At 1.5 T, the total brain volume average was 1,185,283 mm^3^ (SD = 99,426), and at 3 T it was 1,178,542 mm^3^ (SD = 102,826), representing a negligible mean difference (–0.6%) and a small effect size (Cohen's d = –0.07). With ICCs (intraclass correlation coefficients) between 0.978 and 0.993 (Table 3). To visualize agreement and potential systematic bias between 1.5 T and 3 T scanners for selected subcortical volumetric measures, Bland–Altman plots were generated (Figure 3). These plots demonstrate that structure-specific scanner-related differences are particularly evident in deep gray matter regions, where values were minimal and evenly distributed around zero, indicating no significant systematic bias across field strengths. All global volumetric measures demonstrated excellent cross-scanner reliability.

**Table 3:** The intraclass correlation coefficient (ICC) measurements for brain volumes between 1.5 T and 3 T.

ROI	Intraclass correlation coefficient (ICC)
Total brain volume	0.993
Total brain volume without ventricles	0.992
Supratentorial volume	0.992
Supratentorial volume without ventricles	0.992
Total grey matter	0.982
Cerebral white matter volume	0.987
Cortex volume	0.978
Subcortical grey matter	0.980
Thalamus	0.934
Caudate	0.984
Putamen	0.916
Pallidum	0.865
Hippocampus	0.943
Amygdala	0.952
Accumbens area	0.819
Ventral DC	0.921
Corpus callosum (all)	0.927
Corpus callosum (anterior)	0.923
Corpus callosum (mid anterior)	0.791
Corpus callosum (central)	0.760
Corpus callosum (mid posterior)	0.868
Corpus callosum pro (posterior)	0.958
CSF	0.952
3rd ventricle	0.982
4th ventricle	0.988
Lateral ventricles	0.998

### 3.2 Subcortical volumes

Compared with global metrics, several subcortical regions in the deep gray matter exhibited substantial cross-scanner variability. Among these, it was found that the area of the accumbens was the most extreme, showing a 36.1% increase at 3 T compared to 1.5 T (Cohen's d = 1.58; FDR-adjusted *p* = 0.0004; Table 4). This was the most significant relative change, the largest Cohen's d measured among all regions of interest, and likely the largest scanner-related bias present. Though some other subcortical gray matter structures, such as the putamen (+13.1%, d = 1.26), and the amygdala (+10.1%, d = 0.95), also exhibited significant differences (Table 4), the effect of the accumbens area bias was markedly greater. In spite of these systematic biases, the reliability of the rankings of subcortical volumes remained high: ICCs were excellent for the thalamus (0.934), putamen (0.916), and amygdala (0.952), and good for the hippocampus (0.943) and accumbens (0.819) (Table 3).

**Table 4:** Cross-scanner differences: percent change, effect size, and multiple-comparison–adjusted *p*-values.

ROI	1.5 T mean (mm^3^)	3 T mean (mm^3^)	% Change	Cohen's d	Raw p	FDR-p
Total brain volume	1 185 283	1 178 542	−0.57%	−0.07	0.772	0.928
Supratentorial volume	1 042 712	1 043 264	+0.05%	+0.01	0.979	0.979
Cerebral WM volume	482 189	473 591	−1.79%	−0.17	0.462	0.690
Cortex volume	485 827	490 228	+0.90%	+0.11	0.646	0.831
Subcortical GM volume	59 848	62 143	+3.83%	+0.51	0.050	0.134
Thalamus	16 989	15 873	−6.56%	−0.70	0.003	0.021
Caudate	7 053	7 340	+4.07%	+0.32	0.174	0.302
Putamen	9 865	11 157	+13.1%	+1.26	<0.0001	0.0004
Pallidum	4 113	4 117	+0.09%	+0.01	0.970	0.970
Hippocampus	8 301	8 439	+1.66%	+0.22	0.349	0.524
Amygdala	3 142	3 459	+10.1%	+0.95	0.0003	0.003
Accumbens	998	1 359	+36.1%	+1.58	<0.0001	0.0004
Ventral DC	8 329	8 408	+0.95%	+0.10	0.668	0.833
Corpus callosum (All)	3 928	3 689	−6.08%	−0.56	0.017	0.072
Corpus callosum – Anterior	915	994	+8.63%	+0.66	0.008	0.041
Corpus callosum – Mid-Ant	689	583	−15.4%	−1.01	0.0003	0.004
Corpus callosum – Central	716	559	−22.0%	−1.46	<0.0001	0.0004
Third ventricle	858	984	+14.7%	+0.57	0.015	0.065
Fourth ventricle	1 777	1 747	−1.69%	−0.07	0.758	0.928
Lateral ventricles	12 351	13 667	+10.7%	+0.31	0.184	0.310

Percent change is calculated as (3 T – 1.5 T)/1.5 T × 100. Cohen's d is computed from paired data using the pooled SD. False-discovery-rate (FDR)–adjusted *p*-values control for 41 ROI comparisons.

In contrast, other subcortical structures, such as the caudate, pallidum, hippocampus, and ventral diencephalon, showed no significant differences, with all *p*-values exceeding 0.05 after correction (Table 2). The hippocampus, in particular, demonstrated both excellent agreement (ICC = 0.943) and no significant mean-level difference, indicating its robustness across field strengths.

**Figure 4:**
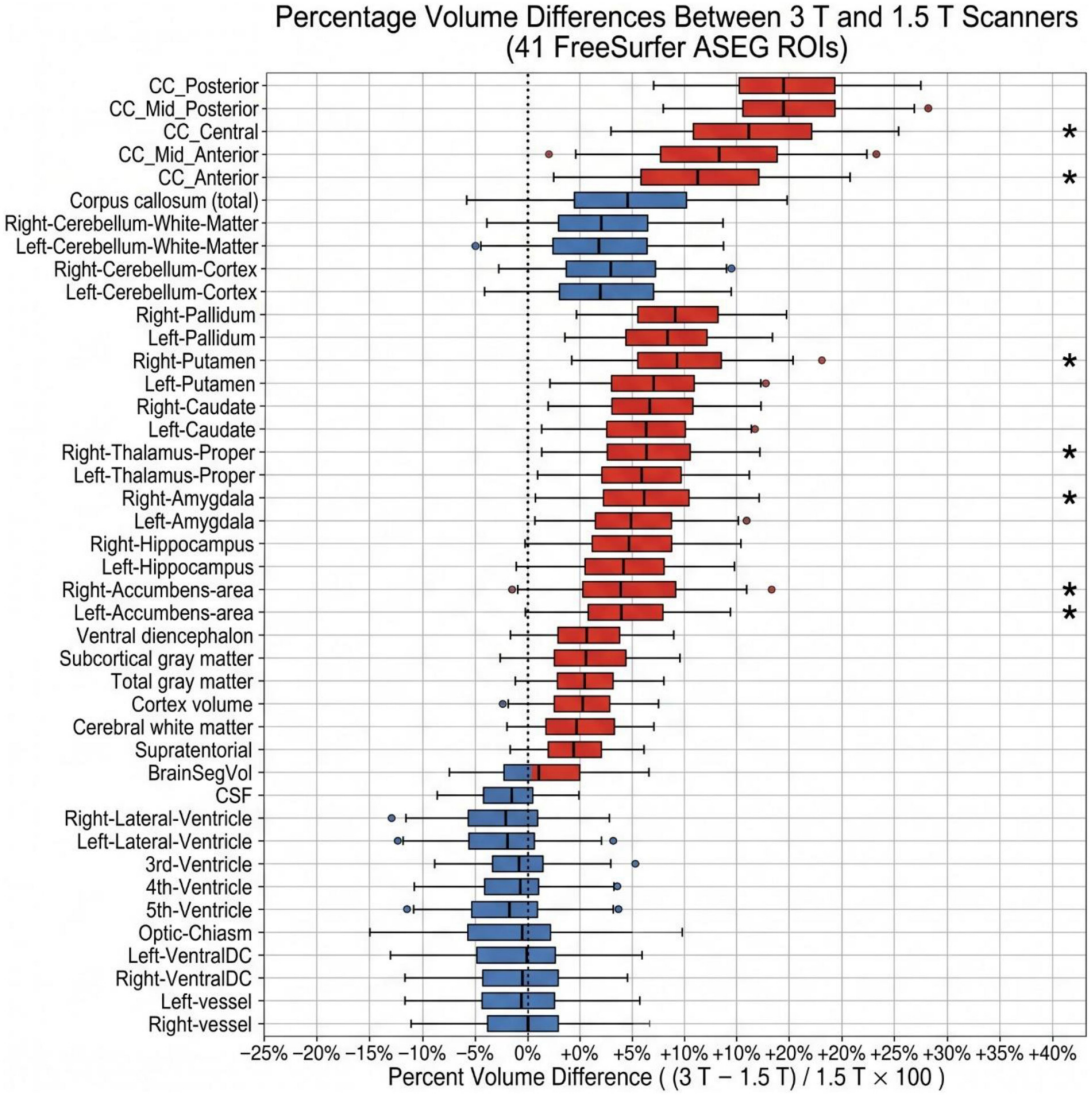
Boxplots of percentage volume differences between 3 T and 1.5 T MRI scanners for 41 FreeSurfer ASEG regions of interest (ROIs). Each boxplot shows the distribution of within-subject percentage differences in volumetric measurements for a bilateral/global ROI, as calculated from paired 3 T and 1.5 T scans of the same individuals. The *x*-axis is scaled from −25% to +40% to reflect the full range of observed differences. The dashed vertical line at 0% indicates perfect agreement between scanners.

The magnitude and direction of percentage volume differences for all 41 brain ROIs between scanners are summarized in a boxplot (Figure 4). This visualization highlights that the most pronounced scanner-related biases occurred in the putamen, amygdala, accumbens, thalamus, and distinct corpus callosum subregions, while global and most cortical measures showed negligible differences.

### 3.3 Percentage differences and effect sizes across ROIs

Table 4 demonstrates the magnitude and statistical significance of cross-scanner differences for selected representative ROIs, while the complete set of all 41 ROIs is provided in Supplementary Table S1. “Percent change, effect Size, and multiple-comparison–adjusted *P*-values,” outlines the magnitude and statistical significance of the 1.5 T vs. 3 T differences for each region of interest (ROI). Global metrics (total brain volume, supratentorial volume, grey and white matter, cortical volume) showed insignificant differences (≤ 1.8%) with very small paired-sample effect sizes (Cohen's d ≤ 0.17). After false-discovery rate (FDR) control for 41 simultaneous comparisons, none of these global metrics were significant (FDR-*p* > 0.90). On the other hand, many deep-grey structures showed substantial effects of scanner-related variability. Putamen (+13.1%, d = 1.26, FDR-*p* = 0.0004), amygdala (+10.1%, d = 0.95, FDR-*p* = 0.003), accumbens area (+36.1%, d = 1.58, FDR-*p* = 0.0004), and thalamus (–6.6%, d = 0.70, FDR-*p* = 0.021) all showed significant differences after adjustment, suggesting the presence of systematic field strength bias. Differences were also observed in sub-regions of the corpus callosum; the central sector decreased by 22.0% at 3 T (d = 1.46, FDR-*p* = 0.0004) while the anterior one increased by 8.6% (d = 0.66, FDR-*p* = 0.041). Ventricular volumes showed moderate percentage changes (third ventricle +14.7%), but none were significant after FDR correction. Overall, Table 5 portrays the majority of whole brain and cortical indices as interchangeable across scanners, while individual subcortical nuclei and callosal sectors show notable, direction-specific bias. The full list of all ROIs incorporated in the FDR correction is shown in Supplementary Table S1.

**Table 5:** Reliability of 1.5 T vs. 3 T volumetric measures: intraclass correlation coefficients with 95% CIs.

ROI	ICC (3,1)	95% CI	Reliability evaluation
Total brain volume	0.993	0.989–0.996	Excellent
Supratentorial volume	0.992	0.987–0.996	Excellent
Total gray matter	0.982	0.969–0.990	Excellent
Cerebral WM volume	0.987	0.979–0.993	Excellent
Cortex volume	0.978	0.962–0.988	Excellent
Subcortical GM volume	0.980	0.966–0.989	Excellent
Thalamus	0.934	0.880–0.964	Excellent
Putamen	0.916	0.845–0.955	Excellent
Pallidum	0.865	0.760–0.925	Good
Hippocampus	0.943	0.896–0.968	Excellent
Amygdala	0.952	0.912–0.975	Excellent
Accumbens	0.819	0.680–0.898	Good
Corpus callosum – Central	0.760	0.583–0.866	Good
Corpus callosum – Mid-Ant	0.791	0.636–0.884	Good
CSF	0.952	0.911–0.975	Excellent
Lateral ventricles	0.998	0.996–0.999	Excellent

Reliability categories follow Cicchetti (1994): Poor < 0.40, Fai*r* = 0.40–0.59, Good = 0.60–0.74, and Excellent ≥ 0.75.

### 3.4 Cross-scanner reliability of volumetric measures

Table 5 summarizes the Reliability of 1.5 T vs. 3 T for the volumetric Measures. The ICC with 95% CIs offers the reproducibility profile across scanners using ICC (3,1). All global measures demonstrated excellent reliability (ICC ≥ 0.75) (ICC = 0.978–0.993) as well as most subcortical ROI thalamus (0.934), putamen (0.916), hippocampus (0.943), and amygdala (0.952) ROI's. The accumbens (0.819) and the central and mid-anterior portions of the corpus callosum (0.760–0.791) exhibited good reliability (ICC = 0.76–0.82). No region fell below the good threshold, which highlights that structures with mean-level bias still retain good rank-order stability across scanners. In conjunction with Table 5, these ICC results lead to the conclusion that efforts to harmonise should improve less reliability and correct systematic offsets in some of the deep-grey and callosal structures.

### 3.5 Corpus callosum and ventricular volumes

The analysis of corpus callosum showed region and bias specific to direction. While one central sector corpus callosum showed a volume decrease of 22% relative to 1.5 Tesla MRI central corpus callosum (+8.6%, FDR-adjusted *p* = 0.041, Table 2), the anterior segment. Good to excellent subregion reliability (ICC = 0.76–0.93; Table 3).

The volume of the third and lateral ventricles showed some percentage changes across the board and these changes (Table 2) did not survive the FDR correction. However, all measurements showed exemplary (ICC) reliability, over 0.95 (Table 3).

### 3.6 Summary of cross-scanner agreement

**Figure 5:**
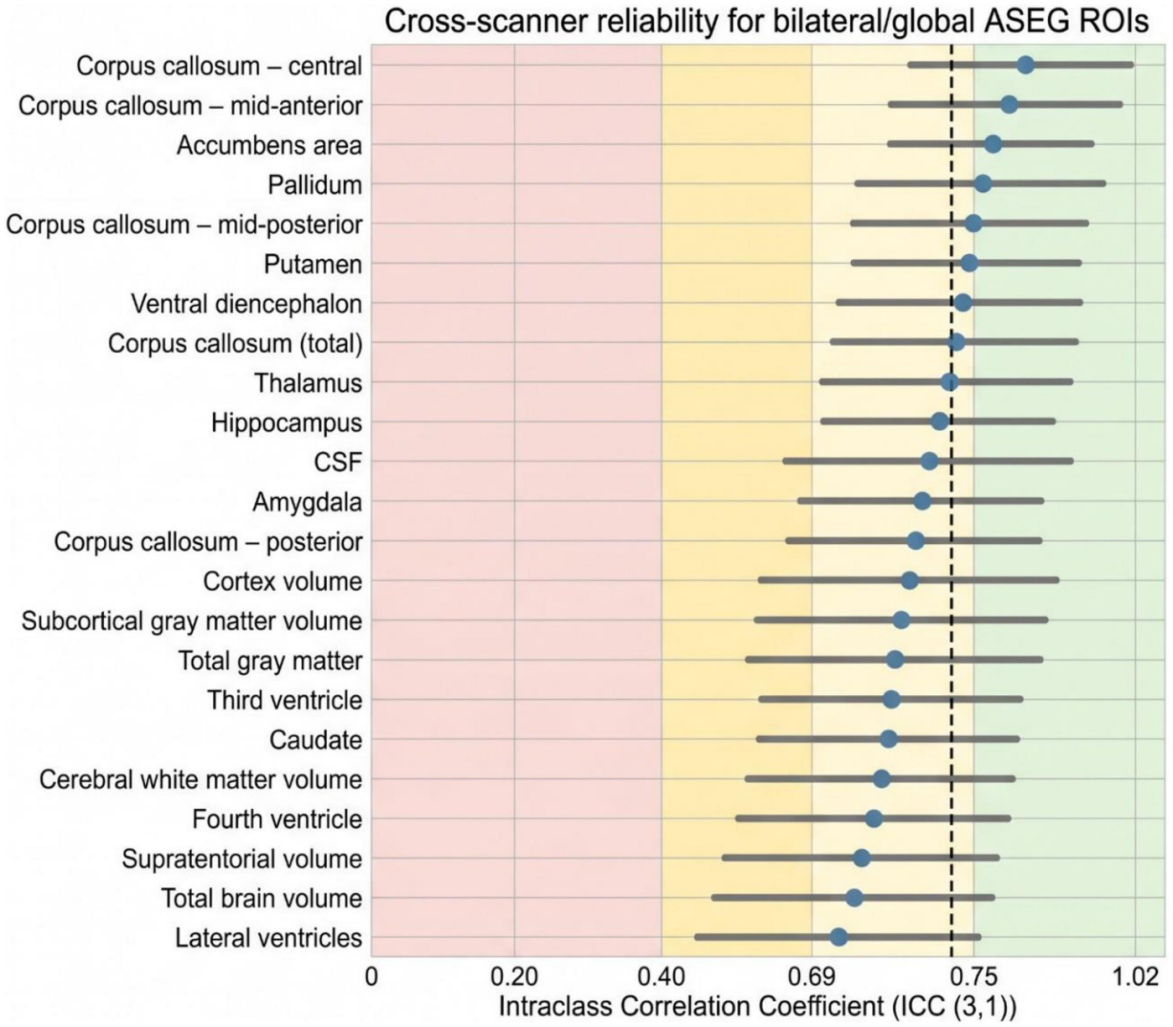
Forest plot of cross-scanner reliability [ICC (3,1)] for bilateral/global ROIs derived from the FreeSurfer ASEG atlas.

Overall, the study found that the global brain and cortical volumetric measures are highly consistent and reliable across 1.5 T and 3 T MRI scanners when standardised protocols and FreeSurfer segmentation are utilized. However, systematic, region-specific biases were observed for select deep-gray nuclei and corpus callosum subregions, despite high rank-order stability. The reliability profile for bilateral/global ROIs derived from the FreeSurfer ASEG atlas was illustrated in a forest plot of ICCs with 95% confidence intervals (CI) (Figure 5). This plot demonstrates that the vast majority of structures exhibit “good” to “excellent” reliability, with only a minority (primarily some corpus callosum sectors) showing lower—but still acceptable—ICC values. These results show the need to focus on subcortical and callosal structures to examine the multi-site or multi-field strength data.

Horizontal gray lines represent the 95% confidence intervals, and blue circles indicate the ICC point estimates for each region of interest (ROI). Background shading denotes reliability categories according to commonly accepted ICC interpretation thresholds: poor (<0.40), fair (0.40–0.59), good (0.60–0.74), and excellent (≥0.75). The dashed vertical line indicates the threshold for excellent reliability (ICC = 0.75). Only bilateral (left + right combined) or whole-structure volumetric measurements are displayed to maintain consistency with Table 5. ICC values demonstrate generally good-to-excellent cross-scanner reliability across the included ASEG-derived ROIs.

## 4 Discussion

A major factor in treating our findings is that they cannot be defined solely by the difference in magnetic field strength. One scanner was a GE 1.5 T system, and the other a Siemens 3 T system. Because scanner strength influences differences in FreeSurfer tissue contrast and segmentation, the GE and Siemens scanners are not the same, even with respect to magnetic field strength. These differences are the combined scanner-related effects reported by FreeSurfer.

The present study examined the reliability of brain volumetric measurements obtained at 1.5 T and 3 T. This question is central to multi-site neuroimaging research, which increasingly integrates datasets acquired across different scanners and field strengths (3, 4, 8). The strong correlations and high ICC values observed for global volumetric measures—including total brain volume, GM, WM, and cortical GM—corroborate earlier findings that these measures are robust across field strengths (6, 10).

Our sample size (*n* = 38) exceeds that of many prior cross-scanner reliability studies (7, 14, 15), enabling more reliable detection of subtle scanner-related effects. Scanning participants on the same day minimized biological variability, meeting recommended standards for test–retest MRI reliability studies (3, 5).

While whole-brain and cortical measures showed excellent agreement, several subcortical nuclei—particularly the thalamus, putamen, amygdala, and accumbens—showed significant differences. These findings are consistent with Chu et al. (7), who also reported reduced reliability in deep gray matter structures. Increased susceptibility effects at higher field strengths, as previously noted by Krasnow et al. (16), may contribute to boundary ambiguity and segmentation discrepancies.

The study found that hippocampus exhibited excellent reliability (ICC = 0.93) with no significant differences between scanners, consistent with Briellmann et al. (17), Chow et al. (18), and Scorzin et al. (19). This confirms that hippocampal volumetry is stable across field strengths and supports its continued use in multi-site Alzheimer's and memory research. In contrast, lower reliability and significant differences in the putamen and thalamus suggest that these structures may require more advanced harmonization, such as ComBat or multi-atlas segmentation. FreeSurfer's segmentation pipeline is known to exhibit field-strength-dependent variations in specific deep nuclei (6, 9). The mixed findings in corpus callosum subregions may reflect sensitivity to local intensity non-uniformity and susceptibility variations.

An unexpected finding of this study is the significant cross-scanner difference evident in the accumbens. The volume increase of +36.1% at 3 T (large effect size, d = 1.58) shows the presence of cross-scanner variance, indicating the structure's scanner-specificity. The volume increase observed for the accumbens greatly exceeds cross-scanner differences observed in other subcortical nuclei. This likely results from the combination of subcortical sensitivity, susceptibility artifacts, sequence-dependent contrast differences, and difficulties of segmentation of small, low-contrast structures. The volume of the accumbens subregion is of critical importance in the study of addiction and psychiatric disorders. In multi-centric or cross-sectional studies, systemic measurement bias is likely to result in significant errors, and the risk is heightened if not managed due to cross-scanner discrepancies. Research in this domain must avoid aggregating data from various studies without adequate synchronization, and scanner disparities necessitate a cautious interpretation of results.

### 4.1 Potential sources of scanner-related bias

Several biophysical and methodological factors may explain why most global metrics remained similar across field strengths while some deep-grey nuclei exhibited large, directionally specific offsets. First, the intrinsic signal-to-noise ratio (SNR) gain at 3 T improves grey/white matter contrast and refines the borders of small, iron-rich structures. This generally results in larger volumes for the putamen and amygdala, a trend we observed (+13% and +10%, respectively) and that corresponds with the inflation reported by Chu et al. (7) and Han et al. (20). However, at higher field strengths, the increased B₀ inhomogeneity and susceptibility artefacts can obscure tissue interfaces or worsen signal voids—especially in high iron content nuclei such as the pallidal and putaminal nuclei—leading to under-segmentation of certain areas (e.g., the thalamus, −6.6% in our data), as noted by Bazin et al. (21) and Langkammer et al. (22).

Second, the two scanners used different vendor-specific implementations of T1-weighted imaging (GE SPGR and Siemens MPRAGE). While voxel size was matched (1 mm^3^), the inversion efficiency, flip angle, and bandwidth of different sequences impact tissue contrast differently; even small alterations in the TR/TI ratios can introduce bias of up to 0.3 mm in cortical-thickness measurements and shift subcortical structures by several percent (23). Our directionally mixed callosal differences (−22% central, +8% anterior) are consistent with Heinen et al. (6), where sequence differences, and not just the field strength, are attributed to region-specific bias. In future studies using harmonized protocols, and preferably factorial designs with both 1.5 T and 3 T systems from all manufacturers, we will be able to address the separate impacts of magnetic field strength and manufacturer.

Cross-scanner agreement is further influenced by the characteristics of the segmentation pipeline. FreeSurfer's probabilistic atlas was predominantly built using 1.5 T data (23); later studies have demonstrated that some of its subcortical labels may be dependent on the field strength, with a decline in thalamic specificity at 3 T (20). Excellent reliability with significant mean-level bias in deep-grey regions is consistent with these studies. The recent deep-learning models FreeSurfer and SynthSeg have shown some increase in reliability across different contrasts and resolutions (23, 24) and may help in reducing the systematic offsets in this study. The combination of our findings and the literature suggests that: (i) higher SNR at 3 T is beneficial for cortex and whole-brain metrics, (ii) contrast-specific biases due to vendor- and sequence-specific factors can be significant for some subcortical nuclei, and (iii) segmentation methods based on different field strengths and vendors are important for achieving full harmonization in mixed-scanner studies.

### 4.2 Implications for multi-centre consortia

Big projects like ADNI, ENIGMA, and the UK Biobank are getting better at combining data from different sites, vendors, and field strengths (22, 25, 26). Our findings demonstrate that researchers can reliably combine 1.5 T and 3 T datasets for analyses centered on global or cortical volumes, contingent upon standardized acquisition protocols and stringent quality control measures. To address significant bias in subcortical structures, statistical harmonization techniques—such as ComBat (27), Bayesian hierarchical models (28), or deep-learning harmonizers (29)—must be employed to rectify systematic offsets prior to pooling. The high ICCs seen even in biased ROIs are interesting because they show that linear scaling can be used for harmonization, since the rank order of subjects stays the same across scanners. However, harmonization approaches were not applied in our study, as the main objective was to quantify inherent scanner-related variability under controlled acquisition and processing conditions.

Overall, our findings indicate that whole-brain and cortical volumes are reliable across scanners, while select subcortical regions require caution when pooling multi-site data. These findings echo earlier calls for harmonized acquisition and post-processing protocols to support multi-center neuroimaging initiatives.

## 5 Conclusion

Global and cortical volumetric measures demonstrated high agreement and excellent reliability between the two MRI systems despite differences in field strength, vendor, and sequence implementation. However, systematic, scanner-related biases reflecting combined effects of field strength, vendor, and sequence were detected in specific deep-grey nuclei (thalamus, putamen, amygdala, accumbens) and corpus-callosum subregions, reaching up to ± 36% despite good-to-excellent reliability. These discrepancies are likely driven by vendor, sequence, and susceptibility differences rather than measurement noise, implying that large-scale or multi-center studies can confidently merge 1.5 T/3 T data for most volumetric endpoints but should apply harmonization or calibration strategies when analyzing the affected subcortical and callosal structures. Future studies utilizing standardized acquisition protocols or factorial designs are necessary to elucidate the distinct influences of field strength, vendor, and sequence.

## 6 Limitations

There are some limitations should be considered and should guide to future work. First, field strength, vendor, and sequence type were all intertwined (GE 1.5 T SPGR and Siemens 3 T MPRAGE), creating limitations in isolating the effects of each. Differences in sequence-specific contrast between SPGR and MPRAGE are known to affect segmentation results without the influence of field strength. Accordingly, the observed differences are due to scanner variability rather than solely to field strength. Second, the utilization of FreeSurfer's longitudinal processing stream. This pipeline was developed to study longitudinal changes within individuals, so it's best used in longitudinal studies with the same scanners to assess its impact on intra-subject variability. It's possible that it led to smaller inter-subject variability in our studies by processing the data more consistently, thereby inflating the ICC metrics. This should be noted because the processing inflates the reported consistencies, consistent with its nature.

Third, with our cohort being neurologically healthy young adults, replication is warranted in disease specific samples (e.g., Alzheimer's disease, multiple sclerosis) as tissue contrast and atrophy patterns will likely differ in pediatric, aging and clinical populations. Third, as we focused only on volumetric measures, cross-scanner assessments on diffusion, quantitative MRI and cortical-thickness as scanner sensitivity are known to be more responsive to variation in scanner are warranted. Lastly, there were clear region-specific reliability issues raised, so future work should aim to investigate and implement more advanced harmonization, such as longitudinal ComBat and to integrate Bayesian hierarchical or deep learning-based image translation, to cross quantitative or multi-echo acquisition to mitigate contrast variability. Considering these areas will be essential to integrating 1.5 T and 3 T data with confidence in differing scanners, sequences and populations in multi-centric neuroimaging consortia.

## Data Availability

The original contributions presented in the study are included in the article/Supplementary Material, further inquiries can be directed to the corresponding author.
