# Supplementary material for: The efficacy of multisite MRI scanners for total brain volume measurements: a cross-sectional study in Saudi Arabia
Source: Front Radiol. 2026 Jun 17;6:1818230. doi: 10.3389/fradi.2026.1818230 (PMC13320661; doi:10.3389/fradi.2026.1818230)
Supplement: Supplementary file 1 [file Datasheet1.pdf]

Supplementary Table S1. Complete ASEG ROI Results (Publication-Ready)

area	Right-VentralDC	Right-vessel	Right-choroid-plexus	5th-Ventricle	WM-hypointensities	Left-WM-hypointensities	Right-WM-hypointensities	non-WM-hypointensities	Le
	3948.5	0	275.5	0	510.6	0	0	0	0
	4265.8	19.8	544.3	0	868.6	0	0	0	0
	3744.9	0	483	0	423.4	0	0	0	0
	3984.7	2.9	639.6	0	930.8	0	0	0	0
	4216.4	7.3	265.1	0	448.7	0	0	0	0
	4024.1	23.8	558.3	0	1095.2	0	0	0	0
	4225.9	16.1	1194.8	0	874.2	0	0	0	0
	3874.4	10.7	877.1	0	1409.2	0	0	0	0
	3953.8	0	502.8	0	830.8	0	0	0	0
	3950.5	10.7	679.9	0	1078.5	0	0	0	0
	3972.7	25.9	450.1	0	459	0	0	0	0
	4251.2	83	549.1	0	1004.1	0	0	0	0
	4591.6	29.8	436.6	0	950.1	0	0	0	0
	4551.6	23.7	686.4	0	1271.2	0	0	0	0
	4119	4.2	475.8	0	572.6	0	0	0	0
	4135.9	7	829.9	0	1152	0	0	0	0
	5224.8	0	315.7	0	579.5	0	0	0	0
	5024.3	3.2	497.4	0	1104.8	0	0	0	0
	3901.8	6.2	268.4	0	340.8	0	0	0	0
	4078.7	11.9	671.3	0	1131.1	0	0	0	0
	4076.9	0	506.3	1.7	785.9	0	0	0	0
	4548.4	40.4	487.6	0	1047.8	0	0	0	0
	4236.2	12.1	432.7	0	524.3	0	0	0	0
	4208.8	18.3	747.3	0	856.4	0	0	0	0
	3469.8	27	395.5	0	455.7	0	0	0	0
	3271.7	56.6	588.8	0	798.1	0	0	0	0
	3683.5	1.9	317.2	0	975.9	0	0	0	0
	3838	12.4	446.7	0	762.7	0	0	0	0
	4265.3	13.8	344.6	0	463.5	0	0	0	0
	4224.9	9.7	644	0	1396.2	0	0	0	0
	5068.8	16.4	456.7	0	588.2	0	0	0	0

area	Right-VentralDC	Right-vessel	Right-choroid-plexus	5th-Ventricle	WM-hypointensities	Left-WM-hypointensities	Right-WM-hypointensities	non-WM-hypointensities	Le
	4866.2	23.3	747.9	0	890.7	0	0	0	0
	3902.6	0	389	0	1647.6	0	0	0	0
	3782.4	8.4	794.9	0	1520.1	0	0	0	0
	3511.9	61.5	262.2	0	516	0	0	0	0
	3751.4	73.5	437.6	0	1359.2	0	0	0	0
	4474.6	22.9	358.9	0	829.9	0	0	0	0
	4087	10.1	622.4	0	1220.1	0	0	0	0
	4512.4	8.6	289.1	0	649.5	0	0	0	0
	4428.4	18.8	523	0	1121.5	0	0	0	0
	4185.4	12.7	560.3	0	717.4	0	0	0	0
	4111.4	7.9	768.2	0	1081.2	0	0	0	0
	4982.4	0	600.2	0	911.7	0	0	0	0
	4637.3	10.4	874	0	1311.3	0	0	0	0
	4135.2	15.1	286.8	0	784.8	0	0	0	0
	4422.2	11.3	479.3	0	1155.4	0	0	0	0
	4149.7	0	441.2	0	1380.6	0	0	0	0
	4325.1	11.9	516.5	0	971.9	0	0	0	0
	4190.9	14	336.6	0	431	0	0	0	0
	4199.1	8.3	646.9	0	989.3	0	0	0	0
	3895.4	29.4	365.6	0	670.9	0	0	0	0
	3933.9	20.5	662.2	0	1085.2	0	0	0	0
	4341.7	0	584.5	0	794.2	0	0	0	0
	4108.6	14.2	707.6	0	1508.3	0	0	0	0
	3976.6	0	463.8	0	490.2	0	0	0	0
	3991.2	9.6	713.9	0	1390	0	0	0	0
	3947.4	0	361.9	0	562.5	0	0	0	0
	3901.8	3.7	711.3	0	858.4	0	0	0	0
	3294.4	7.3	229.5	0	389.2	0	0	0	0
	3758.3	12.2	464.7	0	999.5	0	0	0	0
	3929.7	11.3	247.9	0	706.9	0	0	0	0
	4036.3	12.6	636.1	0	1271.3	0	0	0	0
	4447	22.3	498.7	0	785.9	0	0	0	0
	4534.3	25.3	808.5	0	955	0	0	0	0
	5026.5	12	539.4	0	765	0	0	0	0

area	Right-VentralDC	Right-vessel	Right-choroid-plexus	5th-Ventricle	WM-hypointensities	Left-WM-hypointensities	Right-WM-hypointensities	non-WM-hypointensities	Le
	4643	26.6	895.9	0	1058.6	0	0	0	0
	4155.2	31	449.1	0	1476.1	0	0	0	0
	3759	25.6	691.8	0	1374.7	0	0	0	0
	3807	36.1	383	0	787.5	0	0	0	0
	3841.8	45.4	719.8	0	890.9	0	0	0	0
	3848.5	33.3	831.3	0	653.6	0	0	0	0
	3746.5	48.3	894.7	0	885.6	0	0	0	0
	4264	16	509.5	0	1204.7	0	0	0	0
	4583.7	20	827	0	1640.5	0	0	0	0
	4755	6.6	436.4	0	1187	0	0	0	0
	4349.8	14.5	778	0	1305.4	0	0	0	0
	4387.2	0	372.1	0	417.4	0	0	0	0
	4336.6	19	822.1	0	1063.1	0	0	0	0
